# To announce or not to announce: What is known about the 2016–2017 influenza season in Hong Kong?

**DOI:** 10.1038/emi.2017.76

**Published:** 2017-09-06

**Authors:** Pak-Hin Hinson Cheung, Chi-Ping Chan, Dong-Yan Jin

**Affiliations:** 1 School of Biomedical Sciences, The University of Hong Kong, 21 Sassoon Road, Pokfulam, Hong Kong, China

The death toll of seasonal influenza in Hong Kong in 2017 has hit the headlines and received broad media coverage worldwide. Some commentators compared it with that of severe acute respiratory syndrome (SARS) during the outbreak in 2003. What was it really like? As of 26 August 2017, the influenza activity in Hong Kong had further declined from the peak and returned to the baseline, indicating that the 2016–2017 influenza season is over. From 5 May to 26 August, 586 severe seasonal influenza cases were recorded, including 19 pediatric cases (aged below 18). Among them, 429 adults and 3 children had died. Hospitalization rates were high, especially for the elderly (aged ⩾65). H3N2 was the predominating subtype detected.

It is known that H3N2 is more virulent than other subtypes such as H1N1,^1^ causing more severe infection and higher death rate. It remains to be validated whether the predominant H3N2 viruses in this season have undergone antigenic drifts that reduce vaccine effectiveness, as seen in the 2014–2015 season. It is noteworthy that more than 35% of the H3N2 viruses found in May 2017 in Hong Kong contain N121K mutation in the hemagglutinin (HA) protein, which could render the vaccine less effective.^2^ To what extent this might have exacerbated the severity of this influenza season in Hong Kong merits further investigations. Since 45.4% of the fatal cases are known to have received the influenza vaccine for the 2016–2017 season, the possibility that vaccination cannot effectively protect infection with the circulating H3N2 viruses cannot be excluded.

How does this season compare to the previous ones? The most recent H3N2 season was 2014–2015, whereas H1N1 predominated in 2013–2014 and 2015–2016. Whereas seasonal influenza activity peaked in February and March in the previous three seasons, with an exceptional second wave seen in June 2015, it plateaued in July 2017. Consistent with the notion that H3N2 is more virulent, there were more severe cases and deaths in 2014–2015 than in 2013–2014 or 2015–2016. In contrast, the numbers of severe and death cases in the peak week were similar for 2014–2015 and 2016–2017. The 2016–2017 season is drawing to a close at the end of August; the total number of severe and death cases does not exceed that of 2014–2015 (Table 1). The case fatality was about 2% in both seasons.

The above statistics indicates that the 2016–2017 influenza season in Hong Kong is not more severe than any previous H3N2 season, such as the one in 2014–2015. This was further corroborated by tabulation of the numbers of severe cases by age groups, which indicated a similar pattern for the 2014–2015 and 2016–2017 seasons, in which people aged 50–64 and ⩾65 were most severely affected (Table 1). Underlying diseases and immunosuppression are common in the severe cases in these age groups. The pattern was not seen for the two H1N1 seasons in 2013–2014 and 2015–2016.

Furthermore, the rates for influenza-associated hospitalization, severe cases and deaths in the 2016–2017 season in Hong Kong were generally within the boundaries expected for a H3N2 season and comparable to those in other countries and regions in this and previous H3N2 seasons. The disease burden of seasonal influenza is substantial. As estimated by the Centers for Disease Control and Prevention (CDC), there might have been 9.2–35.6 million cases of influenza-related illnesses, 140 000–710 000 hospitalizations and 12 000–56 000 deaths annually in the United States since 2010. For example, in the H3N2-dominant 2003–2004 season, the estimated number of influenza-associated deaths with underlying respiratory and circulatory causes in the United States was 48 614.^3^ The advanced influenza surveillance system in Hong Kong has just provided a real-life example in support of the estimation made by the CDC.

The Center for Health Protection of Hong Kong has done a superb job in providing near real-time monitoring of seasonal influenza activity in the territory. Starting from the 2014–2015 season, they went one step further to announce their findings, including the numbers of severe seasonal influenza cases and deaths to the general public. This real-time weekly update of influenza activity helps to increase the awareness and vigilance of people against the risk. However, it has also triggered extensive media coverage, including exaggerated description and unfair comparison, which cause panic, anxiety and overreaction from residents, visitors and people from other regions.

Influenza-associated excess deaths with underlying diseases are difficult to track and might not be detected timely by the influenza surveillance system in adjacent regions. Hence, a comparison of numbers between different regions cannot reflect the severity of seasonal influenza activity. Human infection of avian H5N1 and H7N9 viruses has a case fatality of 60% and 40%, respectively. Likewise, about of 10% of cases with SARS and 30% of cases with Middle East respiratory syndrome are fatal. These are emerging viral pathogens with unusual properties. If seasonal influenza is a known evil, they are unknown beasts. Direct comparison of the numbers of deaths caused by seasonal influenza and these emerging viruses is therefore misleading.

Increasing the transparency of influenza surveillance and sharing more information about seasonal influenza activity with the public should not be discouraged. In other words, not to announce is not an option, but the question is how to explain to all stakeholders and educate them about seasonal influenza, emerging microbes and infections. There is much room for improvement in this aspect.

The numbers of severe influenza cases and deaths in the 2016–2017 season in Hong Kong remind us of the significant morbidity and mortality caused by seasonal influenza. Reducing the disease burden and the impact on public hospitals in Hong Kong is our next challenge. The percentage of people in the high-risk groups, including children, the elderly and health professionals, in Hong Kong who receive influenza vaccination is relatively low. Providing more incentives, including more attractive subsidizing schemes, to these groups might be helpful. Prescribing Tamiflu to the elderly who are at higher risk has also been shown to be a safe and effective prophylactic measure. Earlier concerns about the possibility that exposing more people to Tamiflu might accelerate the development of drug resistance can be relieved.^4^ Nevertheless, seasonal influenza will still keep us busy in the years to come. New types of vaccines and antivirals are required in our battle against influenza viruses.

## Figures and Tables

**Table 1:** The number of severe influenza cases in recent seasons in Hong Kong

**Influenza season (number of weeks under study)**	**Predominant subtype**	**Number of severe cases in the peak week**	**Number of severe cases in the season by age group (years)**					**Number of deaths in the peak week**	**Number of deaths in the season**
			**0–17**	**18–49**	**50–64**	**65 or above**	**Total**		
2013−2014 (17)	A/H1N1	32	23	54	73	139	289	22	138
2014−2015 (17)	A/H3N2	74	18	21	65	561	665	56	447
2015−2016 (17)	A/H1N1	44	27	58	133	218	436	18	157
2016−2017 (17)	A/H3N2	75	19	27	89	451	586	56	300

Number of deaths includes deaths in public hospitals only. Original data: http://www.chp.gov.hk/files/xls/flux_data.xlsx.
